# Viral and Bacterial Zoonotic Agents in Dromedary Camels from Southern Tunisia: A Seroprevalence Study

**DOI:** 10.3390/microorganisms10040727

**Published:** 2022-03-29

**Authors:** Simone Eckstein, Rosina Ehmann, Abderraouf Gritli, Mohamed Ben Rhaiem, Houcine Ben Yahia, Manuel Diehl, Roman Wölfel, Susann Handrick, Mohamed Ben Moussa, Kilian Stoecker

**Affiliations:** 1 Bundeswehr Institute of Microbiology (IMB), 80937 Munich, Germany; rosinaehmann@bundeswehr.org (R.E.); manueldiehl@bundeswehr.org (M.D.); romanwoelfel@bundeswehr.org (R.W.); susann.handrick@gmail.com (S.H.); kilianstoecker@bundeswehr.org (K.S.); 2 Veterinary Service, General Directorate of Military Health, Ministry of National Defense, Tunis 1000, Tunisia; raoufgritli@yahoo.fr (A.G.); benrhaiemmohamed@yahoo.fr (M.B.R.); benyahia7280@gmail.com (H.B.Y.); 3 Department of Virology, Military Hospital of Instruction of Tunis, Tunis 1008, Tunisia; mohamed.benmoussa@fphm.rnu.tn

**Keywords:** zoonotic agents, surveillance, monitoring, epidemiology, camels, seroprevalence

## Abstract

The rapid spread of SARS-CoV-2 clearly demonstrated the potential of zoonotic diseases to cause severe harm to public health. Having limited access to medical care combined with severe underreporting and a lack of active surveillance, Africa carries a high burden of neglected zoonotic diseases. Therefore, the epidemiological monitoring of pathogen circulation is essential. Recently, we found extensive Middle East respiratory syndrome coronavirus (MERS-CoV) prevalence in free-roaming dromedary camels from southern Tunisia. In this study, we aimed to investigate the seroprevalence, and thus the risk posed to public health, of two additional viral and two bacterial pathogens in Tunisian dromedaries: Rift Valley fever virus (RVFV), foot-and-mouth disease virus (FMDV), *Coxiella burnetii* and *Brucella* spp. via ELISA. With 73.6% seropositivity, most animals had previously been exposed to the causative agent of Q fever, *C. burnetii*. Additionally, 7.4% and 1.0% of the dromedaries had antibodies against *Brucella* and RVFV, respectively, while no evidence was found for the occurrence of FMDV. Our studies revealed considerable immunological evidence of various pathogens within Tunisian dromedary camels. Since these animals have intense contact with humans, they pose a high risk of transmitting serious zoonotic diseases during active infection. The identification of appropriate countermeasures is therefore highly desirable.

## 1. Introduction

Disease prevalence and epidemiology data acquisition in Africa is severely hampered by a multitude of factors such as the lack of health care infrastructure or funding. While the One Health concept has fostered many international and collaborative projects to improve knowledge of animal and human diseases in Africa, the overall understanding remains very incomplete for many reasons. To start with, Africa as a continent features a multitude of different geographical and climatic microenvironments even on a national scale. Each of these microenvironments fosters conditions for specific microorganisms, vectors and hosts. Furthermore, such microenvironments are strongly influenced by the different traditions and lifestyles of the ethnicities living in them, for example, huge urban centers, agricultural communities and nomadic herders.

**Figure 1:**
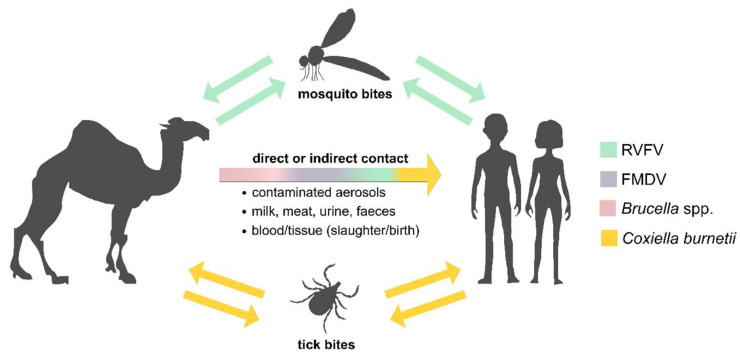
Schematic overview of pathogen transmission pathways. Rift Valley fever virus (RVFV, green), Foot-and-mouth disease virus (FMDV, purple), *Brucella* spp. (pink) and *Coxiella burnetii* (yellow) can be transmitted from dromedary camels both directly and indirectly. Furthermore, RVFV and *C. burnetii* can be transmitted by mosquitos and tick bites, respectively.

In Tunisia, camel breeding is an important agricultural and economic pillar as animals are used for wool or leather, milk and/or meat production as well as for touristic, transport and patrol purposes [1]. However, camels are affected by various infectious diseases. Hence, direct or indirect contact with camels or the consumption of camel products represents a significant source of zoonotic disease transmission to other domestic animals and humans (Figure 1) [2]. Most African dromedary camels kept for milk or meat production roam freely through the desert most of the year, also crossing international borders. This combined with trafficking increases the risk of unnoticedly introducing pathogens from neighboring countries.

Two of the most important bacterial zoonotic diseases associated with domestic livestock are brucellosis and Q fever, caused by the Gram-negative bacteria *Brucella* spp. and *Coxiella burnetii*, respectively. In bovines, small ruminants and camels, an infection with either of these pathogens can lead to abortions, infertility or stillbirth [3,4,5]. Camels are hosts for the two *Brucella* spp. that predominantly affect domestic livestock fertility and human health: *B. abortus* and *B. melitensis* [6]. Infections with both *Brucella* spp. and *C. burnetii* are linked to the consumption of raw milk or meat or direct contact with urine, blood, feces or aborted tissues, especially placentas, or the inhalation of contaminated aerosols [7,8]. Additionally, *C. burnetii* might be also transmitted by ticks (Figure 1) [7]. However, as human brucellosis as well as Q fever lack pathognomonic symptoms but can manifest with fever, headache, skin rash, pneumonia, endocarditis or encephalitis [9,10,11,12,13], infections are often not recognized as such. Moreover, *C. burnetii* and *Brucella* spp. have also been deliberately used against humans in the past as part of active bioweapons programs. Therefore, even today, there is a latent risk of the misuse of these pathogens, e.g., by criminals or terrorists. In this context, active epidemiological surveillance in the event of a disease outbreak makes it easier to distinguish between natural origin and possible deliberate release.

Rift Valley fever virus (RVFV), an arthropod-borne phlebovirus of the *Phenuiviridae* family, is a good example of a pathogen that has to be closely monitored as its geographic distribution is highly influenced by climatic factors and therefore climate change. Mainly transmitted by *Aedes* and *Culex* mosquitoes [14,15], RVFV was first reported by a veterinarian in Kenya in the early 1900s and is widespread in sub-Saharan African regions [14], with increasing reports throughout the African continent and Saudi Arabia [16,17,18,19,20]. There are two transmission cycles for RVFV: a low-level enzootic cycle and an epizootic cycle with fulminant outbreaks causing so-called “abortion storms“ in livestock [21,22,23,24] and considerable pathogenicity in humans [25]. Here, clinical symptoms of the disease can include ocular disease, encephalitis or hemorrhagic fever. Besides mosquitos, exposure to contaminated aerosols or body fluids bears the risk of pathogen transmission to humans (Figure 1). Therefore, increased numbers of human RVFV infections are found in agricultural workers, butchers or domestic livestock keepers [26]. Since dromedary camels are susceptible to RVFV, but only have asymptomatic infections [27], they are a potential source of RVFV transmission to humans. Thus, the seroprevalence of this viral zoonotic agent in Tunisian dromedary camels was investigated in this study.

Another viral zoonotic agent causes one of the most feared and regulated livestock diseases worldwide: foot-and-mouth disease (FMD). Foot-and-mouth disease virus (FMDV), genus *Aphthovirus*, family *Picornaviridae* [28] can be transmitted either directly or indirectly. While human infections are extremely rare [29], FMD is a highly contagious disease in domestic artiodactyl livestock species and can cause enormous economic losses both for the affected farmers (milk loss or increased mortality) but most importantly on the country level due to national and international trading restrictions [30].

In January 2020, we had the rare opportunity to screen a representative set of 500 dromedary camels in southern Tunisia in mostly nomadic small-scale farming for the prevalence of MERS-CoV [31]. Camel farming in Tunisia and the northern Sahara, in general, consists predominantly of the extensive agricultural type with nomadic herding in the desert using the natural resources and relying on a minimum of extra feed provided [32]. In this pastoral herding setting, animals have more individual space, and therefore, artificial effects caused by the crowding of animals in stables or other closed environments are diminished. This in turn effects disease transmission and prevalence. Human intervention with additional feeding, handling of individual animals for breeding or milking is most intensive during the mating and birthing seasons, which coincides with a high transmission risk of the selected pathogens under study. The samples collected here are therefore highly valuable, as they allow insights into the natural distribution of highly pathogenic and zoonotic microorganisms in free-ranging dromedaries and the risk potential of transmission to the herders in an extensive farming style.

## 2. Materials and Methods

### 2.1. Study Design

**Figure 2:**
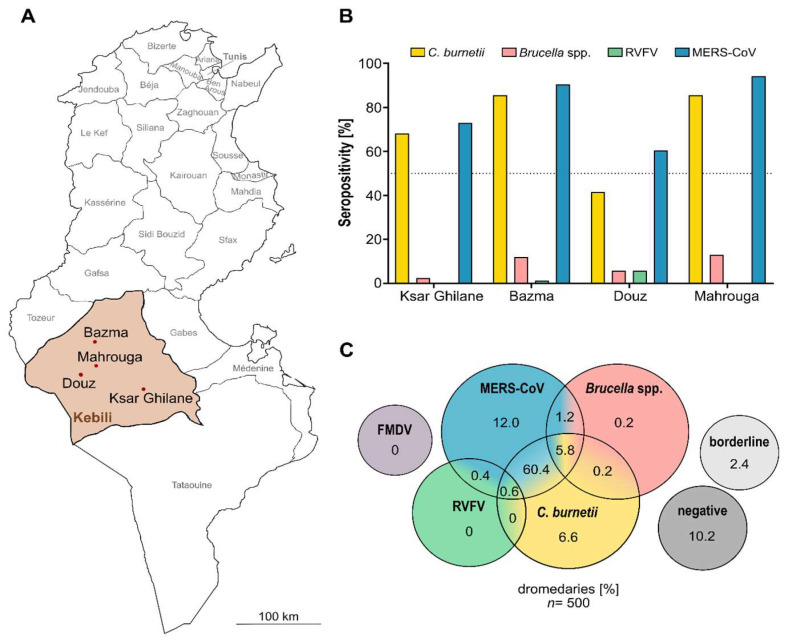
Sampling locations and results of serological screening. (**A**) Map of Tunisia with its different governorates. The four main sampling sites in Kebili (brown): Bazma, Mahrouga, Douz and Ksar Ghilane are highlighted with red dots. (**B**) Seropositivity of animals for the different pathogens sorted by sampling area. (**C**) Venn diagram depicting the ELISA results. Yellow = *Coxiella burnetii*; pink = *Brucella* spp.; green = RVFV; blue = MERS-CoV; purple = FMDV; light gray = borderline; dark gray = negative. Dotted line: 50%. The numbers for MERS-CoV seroprevalence were adapted from our previous study [31].

In this study, sera obtained for an initial MERS-CoV study [31] were re-used to test for additional pathogens. For the previous study, sera were collected from 500 dromedary camels at 20 different sampling sites located around Douz, Mahrouga, Bazma or Ksar Ghilane in the Kebili governorate in southern Tunisia (Figure 2A). At most of the sampling sites (*n* = 13), the camels were kept for milk and meat production, primarily for subsistence use, and allowed to roam the desert freely throughout the year. From these sites, 382 camels were sampled. While attempts were made to evenly collect samples of each age group within a herd, a truly random sample could not be achieved due to logistical constraints and the nature of the herd structures. Furthermore, extensively farmed dromedary herds consist of one sexually mature male (sultan) and its harem (approximately 20–50 sexually mature females and juvenile offspring of both sexes), resulting in a skew of both gender and age distribution. More female animals (*n* = 370) than male ones (*n* = 12) were sampled. Additionally, 118 adult male dromedary camels kept in small, enclosed herds for patrol and transport purposes were sampled. None of the sampled herds were more than 50 km away from the next humid zone, and all of them had regular access to oases.

To collect the sera, camels were caught by the herders and manually restrained. A 4–8 mL blood sample was collected from the jugular vein using an 18-gauge needle into EDTA vacutainer tubes.

The data were analyzed according to the sex (male/female) and age (juvenile: 0–6 months, 6–24 months and adult: 2–6 years, 6–12 years, 12–25 years and >25 years) of the dromedaries as well as sampling location and type of husbandry (free-roaming or enclosed).

### 2.2. Serological Testing

Seropositivity for *Coxiella burnetii* (ID Screen^®^, Q Fever Indirect, IDvet, Grabels, France) as well as *Brucella* spp. (Anti-Brucella-ELISA Kamel IgG, Euroimmun AG, Luebeck, Germany) was analyzed via an indirect ELISA according to the respective manufacturer’s protocol. Anti-FMDV and anti-RVFV IgG was detected via the competitive ELISA PrioCHECK^®^ FMDV NS (Prionics, Lelystad, The Netherlands) and ID Screen^®^ Rift Valley Fever Competition (IDvet, Grabels, France), respectively, using the protocol provided by the manufacturer. All multi-species ELISA have been shown to be valid for camel sera [33,34,35]. Information on semi-quantitative result interpretation for each ELISA is given in Appendix A Table A1. Borderline results were considered negative for all statistical analyses.

### 2.3. Statistical Analysis

For statistical analysis, associations between pathogen seroprevalence in dromedary camels and the study parameters (sex, age, sampling site and animal husbandry) were analyzed. Univariable analysis was conducted by either using Fisher’s exact test or Chi-square test (when necessary). Multivariant analysis was calculated by using multiple logistic regression. Calculations were made using the GraphPad Prism software (Version 8, GraphPad Software, San Diego, CA, USA). *p*-values less than 0.05 were considered statistically significant.

## 3. Results and Discussion

The zoonotic pathogens analyzed in this study have several reservoir species, especially ruminants. Disease prevalence and epidemiology can vary considerably depending on the composition of the different species kept together in the same area. Due to the costs and challenging infrastructure in the desert with small and widely dispersed free-ranging herds, the large-scale vaccination of camels against zoonotic disease agents is not commonly practiced in Tunisia. Currently, dromedary camels are only vaccinated by default against camelpox virus and *Clostridium perfringens*, the causative agent of enterotoxemia. It is therefore still highly desirable to attain a broader coverage of seroprevalence studies on the small-scale level to get a better understanding of both the actual distribution and zoonotic impact of microorganisms as well as their biological properties in different microenvironments.

### 3.1. Majority of Camels Seropositive for Anti-C. burnetii IgG

By screening all 500 dromedary sera for antibodies reactive to *C. burnetii*, we found that, with 73.6% seropositivity, the majority of the sampled dromedary camels were exposed to the pathogen before (Table 1). The respective IgG antibodies were present in at least one camel serum of every sampling site, with the highest seropositivity ratio in Bazma (Figure 2B).

Univariate analysis revealed that the parameters associated with significantly increased seropositivity were adult age, female gender, free husbandry condition and sampling location. By applying the multivariable analysis, however, only adult age, free roaming and the sampling locations Bazma and Ksar Ghilane remained the significant variables (Table 2). Since we only analyzed seropositivity, thus ignoring active infections with the pathogen, the fact that we found more seropositive adult animals than juveniles is conclusive. Furthermore, the significant influence of the husbandry type on seropositivity could be explained by the fact that the risk of introducing new infections is much lower for camels kept enclosed than for animals that wander through the desert freely, thereby encountering other herds or their excrement and tissue remains.

*Coxiellia burnetii* is known to be endemic in Tunisia. However, human Q fever cases are rarely described. While *C. burnetii* has been retrospectively identified as the cause for 9% of hospital admissions [36], only 21 cases of acute Q fever were reported in Tunisia between 2003 and 2007 [37]. Nevertheless, Q fever infections show no pathognomonic symptoms and are therefore often misdiagnosed.

With the exception of one study that reported 44% seropositivity for anti-*C. burnetii* IgG among 534 camel sera [34], little is known about the distribution of Q fever within Tunisian dromedary camel herds. The results of this study revealed seropositivity ratios almost two-fold as high as described before and hence insinuate the extensive circulation of *C. burnetii* within dromedary camels in Tunisia. Several serosurveys from other African countries also found high ratios of positive dromedaries, e.g., in Kenya [38], Saudi Arabia [39] and Egypt [40]. However, the role of camels in shedding *C. burnetii* has not been assessed yet and thus, the question whether dromedaries act as relevant infection reservoirs for humans is a matter of future research.

The extremely low infectious dose of *C. burnetii* and the fact that the camel keepers regularly consume raw camel milk or assist during the birth of new calves offers many interfaces for the transmission of *C. burnetii*. Furthermore, the placentas and tissue remnants are usually left untended in the desert. As *C. burnetii* is a bacterium known for exceptional resistance to environmental stressors and heat compared to other Gram-negative bacteria [41,42], contaminated aerosols could be easily generated and distributed by the constant winds in the desert and inhaled by other livestock or herders. So far, prevalence studies have focused on *C. burnetii* content in milk and blood. Bacterial transmission, however, most consistently occurs via the genital route—especially during birthing season with contact to placenta or amniotic fluid. Therefore, it would be interesting to gather more information on the amount of *C. burnetii* shed in vaginal secretions and placentas of camels [43].

In Tunisia, the vaccination of camels against Q fever is uncommon, as the commercially available vaccine is not licensed for the Tunisian market [44]. Anyway, while clinical signs such as fertility problems and abortion could be reduced completely [45] by, e.g., the off-label use of the vaccine, the prevention of the transmission and excretion of the bacterium is not achieved with the currently available vaccines [46,47]. Furthermore, in the camel farming setting in the desert of southern Tunisia, logistics of transporting and administering vaccines to the widely dispersed small herds is extremely difficult. In principle, vaccination in humans is also possible, as there is one vaccine licensed on the market in Australia [48]. However, as the vaccination can lead to severe adverse reactions in individuals with previous contact to *C. burnetii*, pre-vaccination screening would be required [49]. Thus, other preventative measures such as active surveillance and educational campaigns for the Tunisian population are needed. As even protective materials such as gloves or disinfectants are rare goods in the desert, simple measures such as burying afterbirth remnants in sandpits and heat treatment of camel milk prior to consumption can already considerably reduce the risk of spreading *C. burnetii*.

### 3.2. Low Brucella spp. Seropositivity

With over 500,000 new infections annually, brucellosis is among the most common zoonotic diseases worldwide [50,51]. The serological screening for antibodies against *Brucella* spp. performed in this study revealed 7.4% seropositive animals and therefore no sign of an acute epidemic. However, the 38 seropositive animals provide evidence for the presence of *Brucella* spp. in northern Africa. Univariate analysis showed that the ratio of seropositivity significantly depended on the sampling location (*p* = 0.001) with the majority of seropositive animals located in Bazma and Mahrouga (Table 1, Figure 2B). Since these locations are adjacent areas, our findings may be indicative of a previous series of brucellosis in this region. Furthermore, even though all seropositive camels were adult females (Table 1), no significant correlation between seropositivity and the age (*p* = 0.0647) or sex (*p* = 0.1151) of the animals could be observed. In addition, the type of husbandry did not have a significant impact on seropositivity (*p* > 0.9999). Multiple logistic regression analysis was not possible due to the low count of seropositive animals.

In 1991, an outbreak of brucellosis was reported in the southern governorates of Tunisia, particularly in Gafsa, where 407 cases were diagnosed. As the vast majority of patients had previously consumed raw milk, subsequently performed studies on livestock revealed explosive seropositivity among goats and sheep flocks with positivity rates up to 61% [52]. Although such major outbreaks have not recurred in Tunisia, brucellosis is still endemic there. Over the last years, the number of reported human brucellosis cases per 100,000 inhabitants rapidly increased from <2 in 2013 to almost 9 in 2018 [53]. Similar to Q fever, the number of unreported or misdiagnosed infections is probably high due to the lack of pathognomonic symptoms.

As the predominant *Brucella* species in Tunisia is *B. melitensis*, a practical countermeasure would be the mass vaccination of livestock using the *B. melitensis* vaccine strain Rev.1. However, Rev.1 is a live vaccine and induces abortion in nearly all pregnant females [54]. Like cattle, female dromedaries spend nearly their entire reproductive phase pregnant to produce new offspring and yield milk, which excludes the use of live vaccines. Moreover, vaccination is currently restricted to small ruminants and cattle and would therefore require off-label use [52]. Control and prophylactic measures are very hard to conduct in the Tunisian desert, as access to testing and diagnostic facilities is very limited. Therefore, the possibilities to assess the herd status or acquiring *Brucella*-negative animals are limited. The lack of pathognomonic lesions and symptoms in camels additionally complicates the identification and subsequent elimination of carriers. However, like for *C. burnetii*, contaminated raw milk and meat products are the most frequent routes of *Brucella* spp. transmission. Thus, the heating of milk and thorough cooking of meat before consumption can consistently reduce the risk of brucellosis infections and are easy to apply, even in the desert setting [55].

### 3.3. Sporadic Serologic Evidence of Rift Valley Fever Infections

In this study, only 1% of all camel sera tested positive for antibodies against RVFV, indicating a very low prevalence of the pathogen. Due to the low count of seropositive animals, no statistical analysis was conducted. The five respective seropositive animals were all adult males, kept for transport and patrol purposes at four different sampling sites in Douz (*n* = 4) and Bazma (*n* = 1) (Table 1, Figure 2B), hence showing an isolated pattern of seropositivity in dromedaries in Tunisia. Furthermore, not all animals of one artificial herd had antibodies against the virus. This might be explained by the fact that animals of this type of husbandry are often obtained from livestock markets as adults and not bred by the herders themselves like the herds consisting of one sultan and its harem. Hence, it is most likely that the positive males were already exposed to RVFV before reaching the respective sampling sites.

In 2016, North Africa, especially Tunisia, was identified to be a suitable area for the occurrence and spread of the zoonotic disease Rift Valley fever due to its climate conditions [23]. Shortly after, febrile and non-febrile patients seropositive for antibodies against RVFV confirmed the active circulation of the disease in Tunisia [56]. The first serological evidence of RVFV in Tunisian dromedary camels was then reported by Selmi et al. in 2020, who immunologically analyzed sera collected between 2017 and 2018 from dromedaries within six different governorates. Here, 34% of the tested dromedaries were seropositive for anti-RVFV IgG [27]. Moreover, recent findings also demonstrated the prevalence of specific RVFV antibodies in cattle and sheep from different Tunisian areas [57], evidently supporting the circulation of RVFV in Tunisia.

These transmission patterns are influenced by the biology of the transmitting mosquito species *Aedes* and *Culex*. Female mosquitos produce infected eggs after taking up RVFV during blood meals on infected reservoir hosts. These eggs are very drought-resistant and are primarily deposited in dry basins that are flooded in times of high precipitation [58]. Flooding subsequently leads to mass-hatching events of young mosquitos and severe outbreak events in animals and humans. Such precipitation events can vary considerably in different geographical locations and microenvironments, leading to the periodic appearance of RFV outbreaks. Enzootic transmission is linked to areas with irrigation that support the low-level reproduction of the vectors of RVFV. Therefore, the low seroprevalence found in this study could be linked to the lack of such suitable biotopes in the desert of southern Tunisia.

Low levels of both precipitation and reservoir host density do not favor sustained virus transmission. Whether the seropositive animals in our study were caused by autochthonous infections linked to enzootic transmission near oases, farms and villages or were merely an effect of animal traffic and acquisition from endemic areas such as Egypt remains unanswered. However, Selmi and colleagues found RVFV seroprevalence levels as high as 61% in camels tested in the governorate of Tozeur, which also has a warm desert climate, and 38% seropositivity in Kebili [27]. This apparent incongruency emphasizes the importance of the concept of microenvironments and supports the need for constant vigilance and surveillance to keep track of RVFV epidemiology.

The containment of RVFV spread in endemic areas such as, e.g., African countries can be achieved by vector control via insecticides and the avoidance of unnecessary accumulations of standing water. For livestock, both inactivated and live-attenuated vaccines are commercially available. The latter type is currently applied for vaccinating camels in endemic countries such as Egypt [59]. In Tunisia, however, livestock is currently not vaccinated against RVFV by default due to the lack of documented clinical importance.

### 3.4. No Evidence of Foot-and-Mouth Disease

In the present study, no antibodies against FMDV were found in any of the 500 dromedary sera. No vaccination against FMDV was documented for any of the 500 dromedaries, and as immunization is not financially supported by the government, it is unlikely that any of the animals had been vaccinated against FMDV. Therefore, the absence of seropositivity suggests no previous exposure of the animals to the virus. As FMDV is extremely contagious, this is a strong hint that the Kebili governorate is free of FMD. However, Algeria has experienced outbreaks of FMDV [60], and the open borders and transboundary livestock movements is a constant risk of disease introduction.

Despite several vaccination campaigns and other countermeasures, Tunisia had four major episodes of FMD outbreaks between 1989 and 2017. However, as the FMDV serotypes found in Tunisia are mainly associated with foreign countries, the introduction of FMDV was most likely due to illegal trade [61].

### 3.5. High Serological Evidence for Various Zoonotic Agents in Dromedary Camels of Southern Tunisia

Including the data from our previous MERS-CoV study in the statistics, the majority of dromedaries (87.4%) were seropositive for at least one of the five pathogens (Figure 2C). With 62.6%, more than half of the animals had reactive antibodies against two of the tested zoonotic agents: RVFV + MERS-CoV (0.4%), *C. burnetii* + MERS-CoV (60.8%), *C. burnetii* + *Brucella* (0.2%) and *Brucella* + MERS-CoV (1.2%). Furthermore, with 5.8% (*C. burnetii* + *Brucella* + MERS-CoV) and 0.6% (*C. burnetii* + RVFV + MERS-CoV), 6.4% of dromedary camels sampled were already exposed to three of the five pathogens tested in our studies. Thus, only 12.6% of camel sera were either seronegative for all five pathogens (10.2%) or within the borderline range (2.4%), emphasizing the immense importance of dromedary camels as reservoirs for zoonotic diseases.

## 4. Conclusions

The illegal and uncontrolled trade of animals both at national and transboundary levels leads to a high risk of the introduction of zoonotic pathogens. Combined with a lack of hygiene and disease management strategies, this poses the danger of the sustained transmission of these pathogens between Tunisian camels, but also potentially to their keepers.

In this serosurvey, we found a high seroprevalence of anti-*C. burnetii* IgG in camels from southern Tunisia. Furthermore, we found evidence of *Brucella* spp. and RVFV circulation, albeit the seropositivity was low. In addition, the same set of samples previously revealed an endemic distribution of MERS-CoV in dromedary camels in this region [31]. In total, almost 90% of the tested animals were seropositive for at least one and up to three of the five pathogens we screened for, hence emphasizing the risk of zoonotic disease transmission to humans by their livestock.

For future surveillance studies, we therefore aim to also screen for active infections to address the limitation of our present study. Furthermore, from a biosecurity perspective, knowledge about geographical distribution and phylogeny of different pathogens is mandatory in order to assess the provenance of a pathogen and the likelihood of a bioterroristic background in the event of a local disease outbreak.

Due to the lack of infrastructure and limited options of livestock vaccination and health screening, reducing the pathogen circulation within the herds poses a difficult task. Hence, raising awareness of the presence and the significance of these microorganisms in the local farming community fosters more hygienic handling practices for both humans and animals, thereby reducing the mutual transmission of pathogens.

Easy-to-conduct measures such as the heat treatment of milk and meat products or hygiene management during the birth season with the removal of potentially contaminated afterbirth tissues could have considerable impacts when practiced routinely.

## Figures and Tables

**Table 1:** Seropositive dromedary camels by zoonotic diseases and different sampling parameters. Gray shaded lines: Male animals kept enclosed for transport and patrol purposes.

Sampling Parameter	No. Dromedaries	No. Seropositive Dromedaries (%)		
		*Coxiella burnetii*	*Brucella* spp.	Rift Valley Fever Virus
	500	368 (73.6)	37 (7.4)	5 (1.0)
***Sex***				
**Male**	130	68 (52.3)	5 (3.8)	5 (3.8)
**Female**	370	300 (81.1)	32 (8.6)	0
***Age***				
**Juvenile**	45	13 (28.9)	0	0
0–6 months	4	0	0	0
6–24 months	41	13 (31.7)	0	0
**Adult**	455	355 (78.0)	37 (8.1)	5 (1.1)
2–6 years	80	40 (50.0)	4 (5.0)	0
6–12 years	179	142 (79.3)	18 (10.1)	4 (2.2)
12–25 years	190	167 (87.9)	14 (7.4)	1 (0.5)
>25 years	6	6 (100)	1 (16.7)	0
***Sampling locations***				
**Ksar Ghilane** (∑ = 6)	211	144 (68.2)	5 (2.4)	0
Site 1	28	9 (32.1)	0	0
Site 2	20	7 (35.0)	2 (10.0)	0
Site 3	30	22 (73.3)	0	0
Site 4	20	19 (95.0)	0	0
Site 5	73	54 (74.0)	3 (4.1)	0
Site 6	40	33 (82.5)	0	0
**Bazma** (∑ = 7)	167	143 (85.6)	20 (12.0)	2 (1.2)
Site 1	25	21 (84.0)	0	0
Site 2	30	28 (93.3)	2 (6.7)	0
Site 3	15	14 (93.3)	5 (33.3)	0
Site 4	15	15 (100)	3 (20.0)	0
Site 5	21	18 (85.7)	2 (9.5)	0
Site 6	16	14 (87.5)	4 (25.0)	0
Site 7	45	33 (73.3)	4 (8.9)	2 (4.4)
**Douz** (∑ = 5)	53	22 (41.5)	3 (5.7)	3 (5.7)
Site 1a	4	3 (75.0)	0	1 (25.0)
Site 1b	4	1 (25.0)	0	0
Site 2	24	10 (41.7)	2 (8.3)	1 (4.2)
Site 3	18	7 (38.9)	1 (5.6)	1 (5.6)
Site 4	3	1 (33.3)	0	0
**Mahrouga** (∑ = 2)	69	58 (84.1)	9 (13.0)	0
Site 1	42	35 (83.3)	5 (11.9)	0
Site 2	27	23 (85.2)	4 (14.8)	0
***Animal husbandry***				
**Free-roaming**	382	306 (80.1)	28 (7.3)	0
**Kept enclosed**	118	62 (52.5)	9 (7.6)	5 (4.2)

Bold and italicized headings describe the superior category of the tested variables. Bold subheading describe the respective subordinate categories.

**Table 2:** Univariate and multivariate analysis of anti-*C. burnetii* IgG seropositivity. Borderline results were considered negative for statistical analyses.

Variable	Univariate		Multivariate ^c^	
	OR (95% CI)	*p* Value	OR (95% CI)	*p* Value
***Sex***				
Female	3.91 (2.5–5.94)	<0.0001 (****) ^a^	0.7846 (0.21–2.50)	0.6962 (ns)
***Age***				
Adult (>2 y)	8.74 (4.50–17.71)	<0.0001 (****) ^a^	16.06 (7.49–36.92)	<0.0001 (****)
***Husbandry***				
Free roaming	3.64 (2.356–5.60)	<0.0001 (****) ^a^	14.4 (3.85–61.77)	0.0001 (***)
***Location Site***		<0.0001 (****) ^b^		
Ksar Ghilane			0.35 (0.20–0.61)	0.0002 (***)
Bazma			3.62 (2.10–6.46)	<0.0001 (****)
Douz			0.54 (0.25–1.14)	0.1067 (ns)
Mahrouga			0.93 (0.44–2.10)	0.8481 (ns)

^a^ Fisher’s exact test; ^b^ Chi-square test; ^c^ multiple logistic regression. Bold and italicized headings describe the superior category of the tested variables. **** *p* < 0.0001; *** *p* ≤ 0.001; ns not significant (*p* > 0.05)

## Data Availability

Not applicable.

## Appendix A

**Table A1:** Summary for semi-quantitative result interpretation for all ELISA kits used in this study. For all assays, extinction (OD) was measured at 450 nm. S = sample; Cal = calibrator; NC = negative control; PC = positive control.

ELISA	Result			Formula
	Negative	Borderline	Positive	
Anti-Brucella ELISA camel	Ratio < 0.8	Ratio ≥ 0.8– < 1.1	Ratio ≥ 1.1	$\mathrm{Ratio}=\frac{{\mathrm{OD}}_{S}}{{\mathrm{OD}}_{\mathrm{Cal}}}$
ID Screen^®^ Q Fever Indirect	S/P% ≤ 40%	S/P% > 40%– ≤ 50%	S/P% > 50%	$\frac{S}{P}\%=\frac{{\mathrm{OD}}_{\mathrm{sample}}-{\mathrm{OD}}_{\mathrm{NC}}}{{\mathrm{OD}}_{\mathrm{PC}}-{\mathrm{OD}}_{\mathrm{NC}}}\times 100$
ID Screen^®^ RVF Competition	S/N% > 50%	S/N% < 40%– ≤ 50%	S/N% ≤ 40%	$\frac{S}{N}\%=\frac{{\mathrm{OD}}_{\mathrm{sample}}}{{\mathrm{OD}}_{\mathrm{NC}}}\times 100$
PrioCHECK^®^ FMDV NS	PI = < 50%	/	PI = ≥ 50%	$\mathrm{PI}=100-\left(\frac{{\mathrm{OD}}_{\mathrm{sample}}}{{\mathrm{OD}}_{\mathrm{NC}}}\right)\times 100$

## References

[1] Jemli M.H., Boulaifene H., Azaouzi Z., Ben Salem W., Khaldi S. (2018). Camel breeding development project Tunisia. Rev. Maroc. Sci. Agron. Vét..

[2] Zhu S., Zimmerman D., Deem S.L. (2019). A Review of Zoonotic Pathogens of Dromedary Camels. EcoHealth.

[3] Cunningham B. (1977). The tranfer of Brucella abortus antibodies from dam to calf. Vet. Rec..

[4] Angelakis E., Raoult D. (2010). Q fever. Vet. Microbiol..

[5] Eldin C., Mélenotte C., Mediannikov O., Ghigo E., Million M., Edouard S., Mege J.-L., Maurin M., Raoult D. (2017). From Q Fever to *Coxiella burnetii* Infection: A Paradigm Change. Clin. Microbiol. Rev..

[6] Godfroid J., Scholz H., Barbier T., Nicolas C., Wattiau P., Fretin D., Whatmore A., Cloeckaert A., Blasco J., Moriyon I. (2011). Brucellosis at the animal/ecosystem/human interface at the beginning of the 21st century. Prev. Vet. Med..

[7] Anderson A., Bijlmer H., Fournier P.E., Graves S., Hartzell J., Kersh G.J., Limonard G., Marrie T.J., Massung R.F., McQuiston J.H. (2013). Diagnosis and management of Q fever—United States, 2013: Recommendations from CDC and the Q Fever Working Group. Morb. Mortal. Wkly. Rep. Recomm. Rep..

[8] Wernery U. (2016). Camelid brucellosis: A review. J. Bacteriol. Mycol..

[9] Dalrymple-Champneys W. (1960). The Future of Brucella Infection in Animals and Man. R. Soc. Health J..

[10] Spink W.W. (1956). Brucellosis; epidemiology, clinical manifestations, diagnosis. Semin. Int..

[11] Maurin M., Raoult D. (1999). Q fever. Clin. Microbiol. Rev..

[12] Kaabia N., Letaief A. (2009). Q Fever in Tunisia. Pathol. Biol..

[13] Vanderburg S., Rubach M., Halliday J., Cleaveland S., Reddy E.A., Crump J.A. (2014). Epidemiology of *Coxiella burnetii* Infection in Africa: A OneHealth Systematic Review. PLoS Negl. Trop. Dis..

[14] Daubney R., Hudson J.R., Garnham P.C. (1931). Enzootic hepatitis or rift valley fever. An undescribed virus disease of sheep cattle and man from east africa. J. Pathol. Bacteriol..

[15] Dar O., McIntyre S., Hogarth S., Heymann D. (2013). Rift Valley Fever and a New Paradigm of Research and Development for Zoonotic Disease Control. Emerg. Infect. Dis..

[16] Arthur R., Cope S., Botros B., Hibbs R., Imam I., El-Sharkawy M., Oun S., Morrill J., Shope R., Darwish M. (1993). Recurrence of Rift Valley fever in Egypt. Lancet.

[17] Bukbuk D.N., Fukushi S., Tani H., Yoshikawa T., Taniguchi S., Iha K., Fukuma A., Shimojima M., Morikawa S., Saijo M. (2014). Development and validation of serological assays for viral hemorrhagic fevers and determination of the prevalence of Rift Valley fever in Borno State, Nigeria. Trans. R. Soc. Trop. Med. Hyg..

[18] Durand J.P., Bouloy M., Richecoeur L., Peyrefitte C.N., Tolou H. (2003). Rift Valley Fever Virus Infection among French Troops in Chad. Emerg. Infect. Dis..

[19] Faye O., Bâ H., Ba Y., Freire C.C., Faye O., Ndiaye O., Elgady I.O., Zanotto P.M., Diallo M., Sall A.A. (2014). Reemergence of Rift Valley Fever, Mauritania, 2010. Emerg. Infect. Dis..

[20] Hassan O.A., Ahlm C., Evander M. (2014). A need for One Health approach—Lessons learned from outbreaks of Rift Valley fever in Saudi Arabia and Sudan. Infect. Ecol. Epidemiol..

[21] Bird B.H., Nichol S.T. (2012). Breaking the chain: Rift Valley fever virus control via livestock vaccination. Curr. Opin. Virol..

[22] Chengula A.A., Mdegela R.H., Kasanga C.J. (2013). Socio-economic impact of Rift Valley fever to pastoralists and agro pastoralists in Arusha, Manyara and Morogoro regions in Tanzania. SpringerPlus.

[23] Arsevska E., Hellal J., Mejri S., Hammami S., Marianneau P., Calavas D., Hénaux V. (2015). Identifying Areas Suitable for the Occurrence of Rift Valley Fever in North Africa: Implications for Surveillance. Transbound. Emerg. Dis..

[24] Baudin M., Jumaa A.M., Jomma H.J.E., Karsany M.S., Bucht G., Näslund J., Ahlm C., Evander M., Mohamed N. (2016). Association of Rift Valley fever virus infection with miscarriage in Sudanese women: A cross-sectional study. Lancet Glob. Health.

[25] (2021). Terrestrial Animal Health Code 2021.

[26] Coetzer J.A. (1982). The pathology of Rift Valley fever. II. Lesions occurring in field cases in adult cattle, calves and aborted foetuses. Onderstepoort J. Vet. Res..

[27] Selmi R., Mamlouk A., Ben Said M., Ben Yahia H., Abdelaali H., Ben Chehida F., Daaloul-Jedidi M., Gritli A., Messadi L. (2020). First serological evidence of the Rift Valley fever Phlebovirus in Tunisian camels. Acta Trop..

[28] Gladue D.P., O’Donnell V., Baker-Branstetter R., Holinka L.G., Pacheco J.M., Fernández Sainz I., Lu Z., Ambroggio X., Rodriguez L., Borca M.V. (2013). Foot-and-mouth disease virus modulates cellular vimentin for virus survival. J. Virol..

[29] Capella G.L. (2001). Foot and mouth disease in human beings. Lancet.

[30] Knight-Jones T.J.D., Rushton J. (2013). The economic impacts of foot and mouth disease—What are they, how big are they and where do they occur?. Prev. Vet. Med..

[31] Eckstein S., Ehmann R., Gritli A., Ben Yahia H., Diehl M., Wölfel R., Ben Rhaiem M., Stoecker K., Handrick S., Ben Moussa M. (2021). Prevalence of Middle East Respiratory Syndrome Coronavirus in Dromedary Camels, Tunisia. Emerg. Infect. Dis..

[32] Faye B. (2013). Camel Farming Sustainability: The Challenges of the Camel Farming System in the XXIth Century. J. Sustain. Dev..

[33] Rissmann M., Eiden M., EL Mamy B.O., Isselmou K., Doumbia B., Ziegler U., Homeier-Bachmann T., Yahya B., Groschup M.H. (2017). Serological and genomic evidence of Rift Valley fever virus during inter-epidemic periods in Mauritania. Epidemiology Infect..

[34] Selmi R., Mamlouk A., Ben Yahia H., Abdelaali H., Ben Said M., Sellami K., Daaloul-Jedidi M., Jemli M.H., Messadi L. (2018). *Coxiella burnetii* in Tunisian dromedary camels (*Camelus dromedarius*): Seroprevalence, associated risk factors and seasonal dynamics. Acta Trop..

[35] Yousef M., Mazloum K., Alnakhli H. (2012). Serological evidence of natural exposure of camels Camelus dromedaries to foot and mouth disease virus. Vet. World.

[36] Kaabia N., Rolain J.-M., Khalifa M., Jazia E.B., Bahri F., Raoult D., Letaief A. (2006). Serologic Study of Rickettsioses among Acute Febrile Patients in Central Tunisia. Ann. N. Y. Acad. Sci..

[37] Bellazreg F., Kaabia N., Hachfi W., Khalifa M., Ben Jazia E., Ghanouchi N., Brahem A., Bahri F., Letaief A. (2009). Acute Q fever in hospitalised patients in Central Tunisia: Report of 21 cases. Clin. Microbiol. Infect..

[38] Muturi M., Akoko J., Nthiwa D., Chege B., Nyamota R., Mutiiria M., Maina J., Thumbi S.M., Nyamai M., Kahariri S. (2021). Serological evidence of single and mixed infections of Rift Valley fever virus, *Brucella* spp. and *Coxiella burnetii* in dromedary camels in Kenya. PLoS Negl. Trop. Dis..

[39] El Nabi G., Bakhiet A.O., Alshaikh M.A., Aljumaah R.S., Mohammed O.B., Hussein M.F. (2015). Prevalence of Antibodies to *Coxiella burnetii* in Camel Milk in Riyadh Region, Saudi Arabia: A Comparison with Serum. J. Anim. Res..

[40] Klemmer J., Njeru J., Emam A., El-Sayed A., Moawad A., Henning K., Elbeskawy M.A., Sauter-Louis C., Straubinger R., Neubauer H. (2018). Q fever in Egypt: Epidemiological survey of *Coxiella burnetii* specific antibodies in cattle, buffaloes, sheep, goats and camels. PLoS ONE.

[41] Kersh G.J., Fitzpatrick K.A., Self J.S., Priestley R.A., Kelly A.J., Lash R.R., Marsden-Haug N., Nett R.J., Bjork A., Massung R.F. (2013). Presence and Persistence of *Coxiella burnetii* in the Environments of Goat Farms Associated with a Q Fever Outbreak. Appl. Environ. Microbiol..

[42] Wittwer M., Hammer P., Runge M., Valentin-Weigand P., Neubauer H., Henning K., Mertens-Scholz K. (2022). Inactivation Kinetics of Coxiella burnetii During High-Temperature Short-Time Pasteurization of Milk. Front. Microbiol..

[43] Devaux C.A., Osman I.O., Million M., Raoult D. (2020). Coxiella burnetii in Dromedary Camels (*Camelus dromedarius*): A Possible Threat for Humans and Livestock in North Africa and the Near and Middle East?. Front. Vet. Sci..

[44] OiE Manual of Diagnostic Tests and Vaccines for Terrestrial Animals 2021. Vol. Chapter 3.1.16., Q Fever. https://www.oie.int/fileadmin/Home/eng/Health_standards/tahm/3.01.16_Q_FEVER.pdf.

[45] Vaccins Ceva Tunisian Online Representation. https://www.ceva.tn/Produits/Ovins-Caprins/Vaccins.

[46] De Cremoux R., Rousset E., Touratier A., Audusseau G., Nicollet P., Ribaud D., David V., Le Pape M. (2012). Assessment of vaccination by a phase I *Coxiella burnetii*-inactivated vaccine in goat herds in clinical Q fever situation: 1. FEMS Immunol. Med. Microbiol..

[47] Guatteo R., Seegers H., Joly A., Beaudeau F. (2008). Prevention of *Coxiella burnetii* shedding in infected dairy herds using a phase I C. burnetii inactivated vaccine. Vaccine.

[48] Ruiz S., Wolfe D.N. (2014). Vaccination against Q Fever for Biodefense and Public Health Indications. Front. Microbiol..

[49] Sellens E., Bosward K.L., Willis S., Heller J., Cobbold R., Comeau J.L., Norris J.M., Dhand N.K., Wood N. (2018). Frequency of Adverse Events Following Q Fever Immunisation in Young Adults. Vaccines.

[50] Corbel M.J., World Health Organization, FAO, OIE—World Organisation for Animal Health (2006). Brucellosis in Humans and Animals.

[51] Godfroid J. (2017). Brucellosis in livestock and wildlife: Zoonotic diseases without pandemic potential in need of innovative one health approaches. Arch. Public Health.

[52] Refai M. (2002). Incidence and control of brucellosis in the Near East region. Vet. Microbiol..

[53] Gideon Informatics I., Berger S. (2020). Infectious Diseases of Tunisia. Los Angeles: Gideon Informatics, Incorporated. https://public.ebookcentral.proquest.com/choice/publicfullrecord.aspx?p=6131180.

[54] Blasco J. (1997). A review of the use of B. melitensis Rev 1 vaccine in adult sheep and goats. Prev. Vet. Med..

[55] Van den Heever L.W., Katz K.W., Te Brugge L.A. (1982). On the inactivation of Brucella abortus in naturally contaminated milk by commercial pasteurisation procedures. J. S. Afr. Vet. Assoc..

[56] Bosworth A., Ghabbari T., Dowall S., Varghese A., Fares W., Hewson R., Zhioua E., Chakroun M., Tiouiri H., Ben Jemaa M. (2016). Serologic evidence of exposure to Rift Valley fever virus detected in Tunisia. New Microbes New Infect..

[57] Zouaghi K., Bouattour A., Aounallah H., Surtees R., Krause E., Michel J., Mamlouk A., Nitsche A., M’Ghirbi Y. (2021). First Serological Evidence of Crimean-Congo Hemorrhagic Fever Virus and Rift Valley Fever Virus in Ruminants in Tunisia. Pathogens.

[58] Ngoshe Y.B., Avenant A., Rostal M.K., Karesh W.B., Paweska J.T., Bagge W., Van Vuren P.J., Kemp A., Cordel C., Msimang V. (2020). Patterns of Rift Valley fever virus seropositivity in domestic ruminants in central South Africa four years after a large outbreak. Sci. Rep..

[59] Faburay B., LaBeaud A.D., McVey D.S., Wilson W.C., Richt J.A. (2017). Current Status of Rift Valley Fever Vaccine Development. Vaccines.

[60] FAO Quarterly Reports of the The European Commission for the Control of Foot-and-Mouth Disease (EuFMD). http://www.fao.org/eufmd/resources/reports/quarterlyreport/en/.

[61] Sana K., Ameni B.S., Kaouther G., Jamel C., Samia M., Anissa D., Naceur B.M. (2018). An Overview of Foot and Mouth Disease Situation in Tunisia (1975–2017). J. Vet. Sci. Technol..

